# Impact of Xanthan Gum on the Storage Stability of Pickering Emulsions Stabilized by Myofibrillar Protein Microgel Particles

**DOI:** 10.3390/foods15081398

**Published:** 2026-04-17

**Authors:** Yi Yang, Jingwei Ye, Chenju Zhang, Linjing Gao, Hongbin Lin, Qisheng Zhang, Jiaxin Chen, Rongrong Yu

**Affiliations:** 1 College of Food and Bioengineering, Xihua University, Chengdu 610039, China; 18982615903@163.com (Y.Y.); m18188460632@163.com (C.Z.); 15949321131@163.com (L.G.); hongbin-ok@163.com (H.L.); 2 First Clinical Medical College, Wenzhou Medical University, Wenzhou 325035, China; yjw@wmu.edu.cn; 3 Sichuan Chuanwei Food Industry Innovation Co., Ltd., Meishan 620036, China; bigbeastone@163.com; 4 Chongqing Key Laboratory of Speciality Food Co-Built by Sichuan and Chongqing, Chengdu 610039, China; 5 The First Affiliated Hospital of Wenzhou Medical University, Wenzhou 325000, China

**Keywords:** myofibrillar protein microgel particles, xanthan gum, microscopic morphology, aqueous-phase dispersion characteristics, pickering emulsion stability

## Abstract

Myofibrillar protein microgel particles (MMP) are promising Pickering stabilisers due to their structure and delivery potential. However, their fibrous, irregular shape promotes aggregation, limiting practical use. This study investigated the effect of xanthan gum (XG) concentration (0.025–0.4%) on MMP dispersion in water and its role in stabilising Pickering emulsions. FTIR and interaction analysis revealed that hydrophobic interactions dominate between XG and MMP, followed by hydrogen bonding and electrostatic forces. At higher XG concentrations (0.2–0.4%), complex particle size decreased from 5.21 μm to 4.49 μm, the contact angle increased from 57.67° to 77.33°, and a uniform dispersed state was achieved. Although increasing XG gradually reduced the emulsifying activity of MMP, it significantly improved the emulsion stability. Microstructure analysis showed that at low XG concentrations, emulsions exhibited phase separation. Rheological measurements indicated that XG-MMP complexes increased continuous-phase viscosity and shear resistance, enhancing macroscopic stability. In summary, at a critical XG concentration of 0.2%, the emulsion undergoes a transition from aggregation-driven instability to network-mediated stabilisation, achieved through the interfacial layer with spatial confinement by a weak aqueous-phase network. This work provides a theoretical foundation and a practical design strategy for fabricating highly stable, tuneable Pickering emulsions based on protein microgel particles.

## 1. Introduction

Emulsions are functional systems extensively applied in the food, pharmaceutical, and cosmetic industries [1]. Due to their intrinsic immiscible oil–water composition, they are thermodynamically unstable [2]. During storage or processing, emulsions are susceptible to creaming, flocculation, or coalescence, which can ultimately lead to phase separation. To achieve long-term stability, amphiphilic emulsifiers are commonly incorporated. These compounds adsorb at the oil–water interface, forming a protective layer that reduces interfacial tension and inhibits droplet aggregation [3]. Conventional chemically synthesised surfactants, such as the widely used polyoxyethylene sorbitan fatty acid esters (Tweens), sorbitan fatty acid esters (Spans), and polyglycerol fatty acid esters, have demonstrated excellent emulsifying properties. Nevertheless, growing concerns exist regarding their potential biotoxicity and environmental persistence [4]. Consequently, soluble proteins from natural sources-both animal and plant-based-are considered promising, environmentally friendly alternatives owing to their favourable biocompatibility, biodegradability, and intrinsic surface activity.

Research on the conversion of proteins into micro- and nanogel particles for the stabilisation of Pickering emulsions has recently gained considerable attention. This microgelation approach converts proteins from conventional molecular emulsifiers into highly efficient solid particulate stabilisers. Owing to their biphasic wettability as solid particles, such proteins adsorb irreversibly at the oil–water interface, establishing a dense physical barrier that markedly improves the stability of Pickering emulsions [4,5]. Notably, among various protein sources, muscle-derived proteins demonstrate not only high nutritional quality but also favourable gelling characteristics. These proteins can form three-dimensional network gels upon heating within a relatively low temperature range (55–65 °C), rendering them suitable materials for the fabrication of protein-based microgel particles [6]. However, the dominant protein in muscle-derived systems, myofibrillar protein (MP), possesses an elongated fibrous structure. Under thermally induced conditions, it tends to undergo extensive intermolecular entanglement, leading to the formation of macroscopic continuous gel aggregates rather than discrete microgel particles [7]. Consequently, the practical utilisation rate of muscle-derived proteins remains substantially lower compared to that of plant and dairy proteins.

The top-down preparation of myofibrillar protein microgels (MMP) through mechanical disruption offers a relatively straightforward and efficient method. Sun et al. [8] successfully obtained submicron-scale MMP by means of thermally induced gelation coupled with microfluidisation, which exhibited enhanced emulsifying and interfacial properties relative to native myofibrillar. Subsequently, Feng et al. [9] further investigated the interfacial adsorption characteristics and stability of MMP in various oil-phase systems. Concurrently, studies during the same year examined the influence of environmental stresses, including temperature, ionic strength, and freeze–thaw cycling, on the stability of Pickering emulsions stabilised by MMP. Findings revealed that emulsions were susceptible to demulsification under conditions of low oil content (40–50%), low ionic strength, or following freeze–thaw treatments [10,11]. Moreover, Zhu et al. [12] reported that submicron-scale MMP could be prepared via Mg^2+^-binding combined with high-intensity ultrasonication. While the inclusion of divalent cations markedly improved emulsion stability, the resulting emulsions still displayed discernible phase separation after 3 days of storage. Although the studies cited above have successfully generated nanoscale MMP, it should be noted that MP inherently possesses a filamentous and asymmetrical fibrous architecture, arising from the self-assembly of myosin and actin. As a consequence, even after thermal treatment and homogenisation into microgels, the fundamental structural units retain distinct fibrous and irregular features. Such morphological characteristics provide limited steric hindrance and inadequate mechanical robustness, which can readily promote droplet flocculation. Concurrently, the flexible interfacial film formed is ineffective in suppressing droplet coalescence, leading to an increase in droplet size and ultimately accelerating emulsion phase separation.

Xanthan gum (XG), an anionic microbial polysaccharide, is widely used in the food industry due to its excellent biosafety, stability, and tolerance to high temperatures and extreme pH levels [13]. Studies have shown that XG interacts with proteins via electrostatic, hydrophobic, and hydrogen bonding, filling the continuous phase of emulsions and forming stable network structures. These interactions significantly enhance the stability of various protein-based emulsions, including those derived from plant and animal sources, while conferring considerable tolerance to variations in system pH and ionic strength [14,15]. Existing studies have investigated the mechanisms by which XG inhibits the adverse interaction between MP and gallic acid, as well as the mechanisms by which it improves the gelling and emulsifying properties of MP [16,17]. Furthermore, research has examined the effect of XG on the structural modification of MP under high-pressure treatment, and the influence of ultrasonic treatment on the physicochemical properties of stable MP-XG emulsions [18,19]. These studies confirm that XG enhances the gelling and emulsifying properties of MP by modifying its structure and regulating the microstructure of the emulsion.

However, although numerous studies have reported on the interaction between MP and XG and their applications in emulsion systems, there remains a lack of systematic understanding regarding the mechanism by which MMP and XG, which possess inherent fibrous or irregular structures, synergistically stabilise Pickering emulsions. In particular, the influence of XG concentration on the microstructure, interfacial properties, rheological behaviour, and overall stability of MMP-stabilised emulsions, as well as the underlying mechanisms, remains unclear. Based on the structural characteristics of MMP and XG, we hypothesise that XG forms a three-dimensional gel network within the aqueous phase, which engages in spatial entanglement or electrostatic attraction with MMP. This interaction is proposed to generate a denser composite network, thereby enhancing steric hindrance effects and improving the mechanical strength of the interfacial film. To test this hypothesis, the present study first characterised XG-MMP composite systems across a range of XG concentrations (0–0.4%, *w*/*v*). Measurements included FTIR spectra, intermolecular forces, particle size distribution, zeta potential, contact angle, and scanning electron microscopy (SEM). Subsequently, Pickering emulsions were prepared using these composites to investigate the effect of XG concentration on emulsion stability and rheological properties, to elucidate the transition from instability to stability, and to determine the critical XG concentration. This work provides meaningful insights and potential strategies for mitigating migration, flocculation, and coalescence issues arising from irregular protein microgel particles within Pickering emulsion systems.

## 2. Materials and Methods

### 2.1. Materials and Reagents

Fresh pork tenderloin was obtained from a local Walmart supermarket (Chengdu, China). Refined soybean oil was supplied by Shanghai Fulinmen Food Co., Ltd. (Shanghai, China). XG was purchased from Shanghai McLean Biochemical Technology Co., Ltd., (Shanghai, China). All other chemicals and reagents used were of analytical grade.

### 2.2. Extraction of MP

MP extraction was performed following the method of Li and Zhang et al. [20,21]. All extraction steps were carried out at 4.0 °C. Briefly, fresh pork tenderloin was trimmed of visible fat and connective tissue, then minced into small pieces. The mince was homogenised in phosphate-buffered solution (20 mM phosphate, 0.1 M NaCl, 2.0 mM MgCl_2_, 1.0 mM EGTA, pH 7.0) at a ratio of 1:4 (*w*/*v*) for 3 min at 8000 rpm. The homogenate was centrifuged at 3500× *g* for 15 min at 4.0 °C, and the pellet was retained. This homogenization–centrifugation cycle was repeated twice. The resulting pellet was then re-homogenised with four volumes of 0.1 M NaCl solution and centrifuged again under the same conditions; this procedure was performed once. Subsequently, the pellet was homogenised with four volumes of phosphate buffer (pH 6.0), filtered through gauze, and centrifuged to recover the MP precipitate. The MP concentration was determined using the biuret method.

### 2.3. Preparation of XG-MMP Complexes

The preparation of MMP was carried out according to the method of Sun et al. [8], with slight modifications. The extracted MP paste was dissolved in phosphate buffer (pH 5.5, 0.1 M NaCl) to form a 2% (*w*/*v*) MP solution, which was first homogenised for 2 min at 10,000 rpm in an ice-water bath. The solution was subsequently magnetically stirred in a water bath at 60 °C for 15 min. After cooling to ambient temperature, it was subjected to three passes of high-pressure homogenisation at 40 MPa. The pH was then adjusted to 7.0 using 1 M NaOH to obtain the final MMP suspension. The prepared MMP solution was centrifuged, and a portion of the supernatant was removed to obtain a 4% (*w*/*v*) MMP solution. This concentrated MMP solution was then mixed in a 1:1 ratio with XG solutions of different concentrations (0%, 0.05%, 0.1%, 0.2%, 0.4%, and 0.8% *w*/*v*). The mixtures were magnetically stirred for 3 h to facilitate protein-polysaccharide binding. This procedure yielded XG-MMP solutions. The final concentration of MMP was fixed at 2% (*w*/*v*), while that of XG varied at 0%, 0.025%, 0.05%, 0.1%, 0.2%, and 0.4% (*w*/*v*).

### 2.4. FTIR of XG-MMP Complexes

FTIR spectra of the XG-MMP complexes were recorded according to the method of Guo et al. [1]. A freeze-dried sample (1 mg) was mixed with potassium bromide (KBr, 100 mg), finely ground and compressed into a pellet. Spectral acquisition was performed using an FTIR spectrometer (Nicolet iS10, Thermo Fisher Scientific, Waltham, MA, USA) over the range of 400–4000 cm^−1^.

### 2.5. Intermolecular Interaction Forces of XG-MMP Complexes

The intermolecular forces within the MMP-XG complexes were characterised according to the method of Zhang et al. [22]. Four solvent systems were prepared: S_1_ (0.05 mol/L NaCl), S_2_ (0.6 mol/L NaCl), S_3_ (0.6 mol/L NaCl containing 1.5 mol/L urea) and S_4_ (0.6 mol/L NaCl containing 8 mol/L urea). The XG-MMP complexes were diluted into each solvent at a 1:5 (*v*/*v*) ratio, vortexed thoroughly to ensure complete mixing, and incubated at 4 °C for 1 h. After centrifugation (8000× *g*, 10 min), the protein content in the supernatant was determined using the Lowry method.

(1) $$\mathrm{Electrostatic}\;\mathrm{interaction}=S_{2}-S_{1}$$

(2) $$\mathrm{Hydrogen}\;\mathrm{bonds}=S_{3}-S_{2}$$

(3) $$\mathrm{Hydrophobic}\;\mathrm{interactions}=S_{4}-S_{3}$$

### 2.6. Particle Size and Zeta Potential of XG-MMP Complexes

The volume mean particle diameter (D_[4,3]_) of the XG-MMP complexes was determined using a laser diffraction particle size analyser (Bettersize 2600, Bettersize Instruments Ltd., Dandong, China), following a procedure adapted from Li et al. [23]. The refractive indices for the dispersed phase and the dispersant were set at 1.520 and 1.333. The zeta potential of the complexes was assessed with a Nano-ZS Zetasizer (Malvern Instruments Ltd., Worcestershire, UK), with equilibration maintained at a constant temperature of 25 °C for 120 s; all samples were diluted 100-fold with deionised water (to avoid multiple scattering effects) [14].

### 2.7. Contact Angle of XG-MMP Complexes

Following the procedure outlined by Li et al. [24], the contact angle (θ) of the XG-MMP complexes was determined using an optical contact angle metre (SL200L1, KINO Scientific Instrument Inc., Boston, MA, USA). For the measurement, 0.2 g of the freeze-dried complexes was compressed into thin sheets (1.5 mm × 12 mm). A 5 μL water droplet was dispensed onto the sheet surface, and then the morphology of the droplets was imaged.

### 2.8. Microstructure of XG-MMP Complexes

Based on the methodology of Ren et al. [14], freeze-dried XG-MMP complexes were fixed, sputter-coated with gold, and subsequently imaged using a scanning electron microscope (SEM3200A, Guo Yi Quantum Science and Technology Co., Hefei, China). The observations were conducted at an acceleration voltage of 10.0 kV and a magnification of 200×. Following the method described by Matsuyama et al. [25], the microstructure of the XG-MMP complexes was further examined using cryo-scanning electron microscopy (Cryo-SEM, SU-8100, Hitachi, Ltd., Tokyo, Japan). Briefly, samples were rapidly cryo-fixed by immersion in liquid nitrogen, and subsequently transferred under vacuum into the preparation chamber via a cryogenic transfer system. Within the preparation chamber, sublimation was carried out at −90 °C, followed by sputter coating. Finally, the samples were transferred to the Cryo-SEM chamber and imaged at an acceleration voltage of 3 kV.

### 2.9. Emulsion Preparation

The emulsion was prepared by homogenising the XG-MMP complexes solution (described in Section 2.3) with soybean oil at a 9:1 (*v*/*v*) ratio for 2 min at 15,000 rpm using a high-shear homogeniser. The resulting emulsion was stored at 4 °C and analysed within 24 h or after 30 days.

### 2.10. Emulsification Properties

Following the method of Song et al. [26], the freshly prepared emulsion (50 μL) was mixed with 0.1% sodium dodecyl sulphate (SDS) solution (10 mL). Absorbance at 500 nm was measured immediately (A_0_) and again after 10 min (A_10_). The emulsion activity index (EAI) and emulsion stability index (ESI) were calculated using the following equations:

(4) $$\mathrm{EAI}(m^{2}/g)=\left(2\right.\times 2.303\times A_{0}\times N)/[c\times \left(1-\varphi \right)\times 10^{4}]$$

(5) $$\mathrm{ESI}\left(\%\right)=A_{10}/A_{0}\times 100$$

where N is the dilution factor (N = 200), c represents the protein concentration (g/mL), φ denotes the volume fraction of the oil phase in the emulsion (10%), and A_0_ and A_10_ correspond to the absorbance values measured immediately (0 min) and after 10 min, respectively.

### 2.11. Measurement of Emulsion Particle Size and Potential

The particle size of the emulsions was measured using a laser diffraction particle size analyser (Bettersize 2600, Bettersize Instruments Ltd., Dandong, China), following a procedure adapted from He et al. [27]. The refractive indices for the dispersed phase and the dispersant were set at 1.475 and 1.333. For zeta potential determination, the emulsions were diluted 100-fold prior to analysis using a Nano-ZS Zetasizer (Malvern Instruments Ltd., Worcestershire, UK) [2].

### 2.12. Emulsion Appearance Morphology

Freshly prepared emulsion samples (15 mL) were placed into glass bottles and stored at 4 °C. The emulsions were photographed, and the height of the emulsified layer was determined on days 0 and 30. The emulsification index (EI) was calculated according to the method described by Phosanam et al. [2]:

(6) $$\mathrm{EI}\left(\%\right)=H_{0}/H_{T}\times 100$$

where H_0_ is the height of the clear liquid layer and H_T_ is the total height of the emulsion.

### 2.13. Emulsion Microscopic Morphology

#### 2.13.1. Cryo-Scanning Electron Microscopy (Cryo-SEM)

Cryo-SEM images of the emulsion were obtained according to the method described in Section 2.8.

#### 2.13.2. Optical Microscope

The microstructure of the emulsion was examined using an optical microscope (ZL2800T, Aos Microoptical Instruments Co., Ltd., Shenzhen, China) [28]. A 20 µL aliquot of the emulsion was placed onto a microscope slide and imaged using the microscope’s integrated display system.

#### 2.13.3. Confocal Laser Scanning Microscopy (CLSM)

The microstructure of the emulsion was visualised using CLSM (CLSM 600, Sunny Instrument Co., Ltd., Ningbo, China) according to an established protocol Cai et al. [29], with slight modifications. Before observation, 1 mL of the emulsion was stained with 20 μL of 0.1% Nile Red aqueous solution (oil-phase) and 20 μL of 0.1% Nile Blue methanol solution (protein). After thorough mixing, samples were incubated in the dark for 30 min. A 20 μL volume of the stained emulsion was then placed on a microscope slide and examined under the CLSM using a 20× objective. Nile Red and Nile Blue were excited at 488 nm and 633 nm, respectively.

### 2.14. Rheological Properties

The rheological properties of the emulsions were characterised using a dynamic shear rheometer (MCR302, Anton Paar GmbH, Graz, Austria) equipped with a 50 mm parallel-plate geometry and a fixed gap of 1.0 mm. All measurements were performed at 25 °C. Following the methodology of Fan et al. [30], three distinct scanning modes were employed: steady shear, amplitude sweep, and frequency sweep.

Steady shear tests were performed over a shear-rate range of 0.1–100 s^−1^ to determine the apparent viscosity (η) of the emulsion. Amplitude sweeps were conducted at a fixed frequency of 1 Hz, with the strain varying from 0.1% to 100%. Frequency sweeps were carried out within the linear viscoelastic region (maintained at a constant strain of 1%) over an angular frequency range of 1–100 rad·s^−1^.

### 2.15. Statistical Analysis

All experiments were conducted in at least triplicate. Data are expressed as mean ± standard deviation. SPSS 25.0 (SPSS Inc., Chicago, IL, USA) and one-way ANOVA followed by Duncan’s test were employed for statistical analysis and significance assessment (*p* < 0.05) separately. Graphs and further data processing were carried out using Origin 10.2 (Origin Lab, Northampton, MA, USA).

## 3. Results and Discussions

### 3.1. Characterisation of XG-MMP Complexes

#### 3.1.1. FTIR Analysis

**Figure 1:**
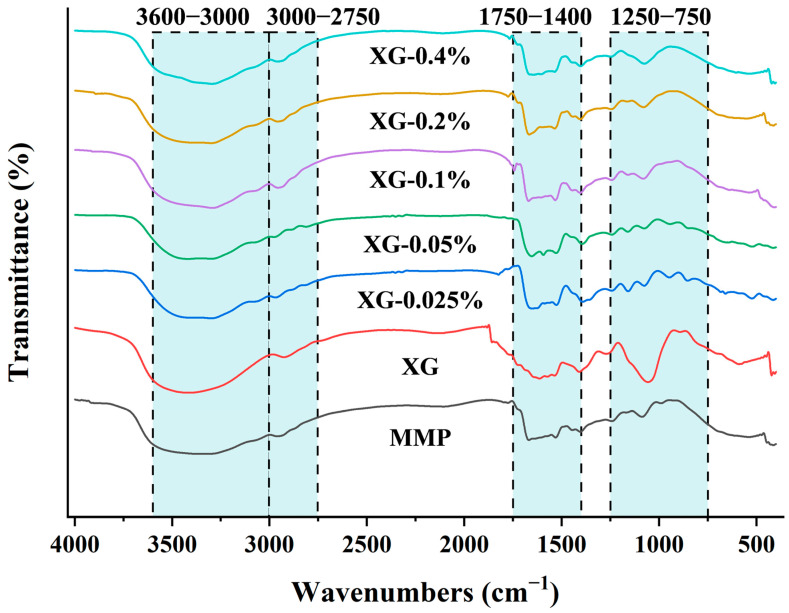
FTIR spectra for MMP (control) and a series of XG-MMP complexes. Abbreviation: MMP, myofibrillar protein microgels; XG, Xanthan gum.

FTIR spectroscopy was employed to analyse the chemical structures and functional groups of the complexes, thereby elucidating the nature of their interactions. As presented in Figure 1, upon mixing XG and MMP, notable alterations in the FTIR peak profiles were observed, indicating substantive interaction between the two components and suggesting the formation of a complex rather than a simple physical mixture. Characteristic shifts were identified within four key spectral regions: 3000–3600 cm^−1^ (O-H stretching vibration), 2750–3000 cm^−1^ (C-H stretching vibration), 1400–1750 cm^−1^ (stretching vibrations of -COO- and C=O bonds), and 750–1250 cm^−1^ (stretching vibrations of C-O-C, C-O-H, and C-C bonds) [31].

The region between 750 and 1250 cm^−1^, commonly termed the “polysaccharide fingerprint zone”, is dominated by stretching and bending vibrations of bonds such as C-O-C, C-O-H, and C-C, and is highly sensitive to polysaccharide configuration, conformation, and intra- or intermolecular interactions. With increasing XG concentration, the spectral profile in this region evolved from multiple discrete peaks into a smoother, dominant peak, suggesting that interactions with MMP induced a reorganisation of the local ordered structure and hydrogen-bonding network within the polysaccharide chains.

The 1600–1750 cm^−1^ region is typically employed to monitor protein secondary structure. However, the secondary structure of protein within the MMPs is largely “locked” due to gelation, making it difficult for FTIR to detect subtle conformational changes inside the rigid gel matrix. Hence, the observed spectral changes in this region are primarily attributed to alterations in the microenvironment of surface-exposed carbonyl groups (C=O). This implies that XG molecules may form hydrogen bonds with surface C=O groups on MMP via their hydroxyl and other functional groups, thereby modulating the vibrational behaviour of these surface moieties. Concurrently, slight shifts in the carboxylate vibration bands of XG near −1605 cm^−1^ and −1415 cm^−1^ further support that the carboxyl groups of XG may form hydrogen bonds with the polar groups on the surface of MMP. In many studies of polysaccharide-protein complexes, hydrogen bonding between carboxyl groups and polar groups is a common mechanism of interaction; research has shown that the formation of MP-chitosan complexes and the interaction between soya protein isolate and XG are both related to hydrogen bonding [32,33].

Furthermore, as the XG concentration increased, the intensity of the characteristic peak near 2954 cm^−1^ steadily rose, and the O-H stretching vibration progressively shifted toward 3283 cm^−1^, indicating that both hydrogen bonding and hydrophobic interactions contribute to the formation of the XG-MMP complex [34]. These findings align with the structural characterisation of soy protein isolate-XG complexes reported by Li et al. [35].

#### 3.1.2. Intermolecular Interactions Analysis

**Figure 2:**
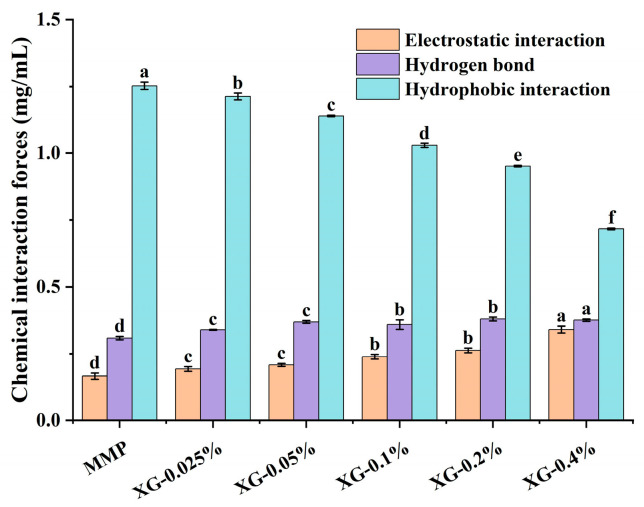
Intermolecular interactions for MMP (control) and a series of XG-MMP complexes. Different lowercase letters (a–f) indicate significant differences between XG concentrations *(p* < 0.05).

As shown in Figure 2, the dominant interaction among MMP molecules themselves is hydrophobic, followed by hydrogen bonding and electrostatic forces. This is likely attributable to the thermal denaturation and subsequent gelation of the protein, during which hydrophobic regions become exposed, promoting hydrophobic-driven aggregation. With increasing XG concentration, electrostatic repulsion and hydrogen bonding within the XG-MMP system become progressively stronger, whereas hydrophobic interactions decrease significantly (*p* < 0.05). The enhancement of electrostatic effects can be ascribed to the anionic nature of XG, which increases the overall negative charge density and thereby strengthens electrostatic repulsion between the complexes [36]. Concurrently, the rise in hydrogen-bonding contributions suggests the establishment of stable hydrogen bonds between hydroxyl groups of XG and complementary sites on MMP, a finding consistent with the FTIR spectral analysis. Notably, hydrophobic interactions remained the predominant attractive force within the composite system, albeit with an overall declining trend. This pattern suggests that at higher concentrations, XG may penetrate the porous structure of the MMPs, competitively occupying the internal hydrophobic region [22].

#### 3.1.3. Appearance, Particle Size, and Zeta-Potential Analysis

**Figure 3:**
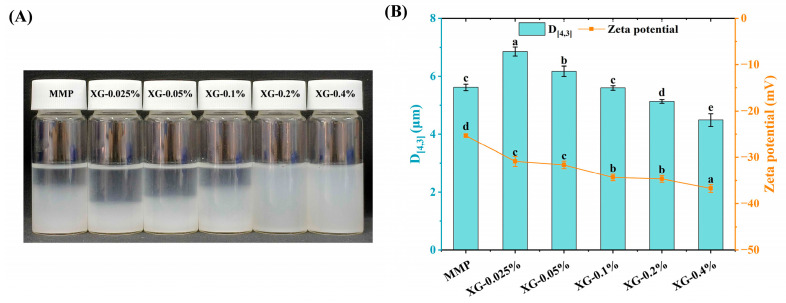
Appearance (**A**), particle size and zeta-potential (**B**) for MMP (control) and a series of XG-MMP complexes. Different lowercase letters (a–e) indicate significant differences between XG concentrations (*p* < 0.05).

The visual appearance of the XG-MMP solutions is presented in Figure 3A. After the solutions were left to settle for 2 h, both the control (MMP alone) and low-XG-concentration samples exhibited varying degrees of phase separation. Notably, the addition of low concentrations of XG induced more pronounced flocculation and precipitation compared to the control. As the XG concentration increased, the composite solutions became progressively more stable, showing no visible stratification. As shown in Figure 3B, the volume-mean particle size increased significantly to 6.86 µm upon addition of 0.05% XG (*p* < 0.05). With further elevation of XG concentration, the particle size gradually decreased, reaching 4.91 µm at the 0.4% XG concentration (*p* < 0.05). Overall, the particle size of all XG-MMP complexes remained around 5 µm. In parallel, the absolute zeta-potential of the complexes rose with increasing XG content, attaining a maximum of 37.8 mV at 0.4% XG (*p* < 0.05).

At low XG concentrations, the limited number of XG chains may adsorb simultaneously onto multiple MMPs, thereby aggregating them into larger flocs that settle readily [11,37]. Although the carboxyl groups of XG are ionised in aqueous solution, enhancing electrostatic repulsion within the system, the formation of large aggregates remains the dominant factor driving instability [38]. Conversely, at high XG concentrations, a surplus of XG chains could fully adsorb onto and penetrate the porous structure of MMP particles. This creates a steric barrier that hinders the close approach of composite particles, while the increased surface charge further strengthens inter-particle electrostatic repulsion, collectively conferring long-term stability to the system [39].

#### 3.1.4. Contact Angle and SEM Analysis

**Figure 4:**
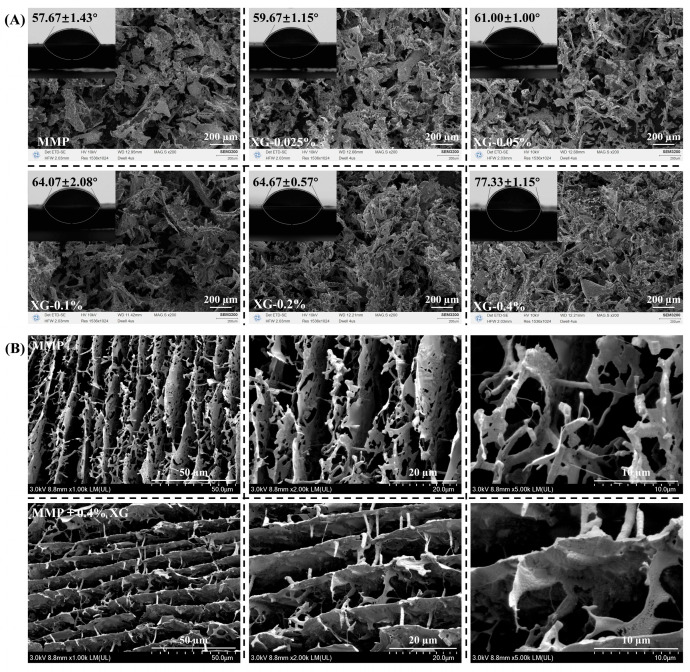
Contact angle, SEM images (**A**) and Cryo-SEM images (**B**) for MMP (control) and a series of XG-MMP complexes. The results for the contact angle are expressed as the mean ± standard deviation.

The water contact angle (θ) was employed to assess the wettability of XG-MMP complexes at the oil–water interface. Particles exhibiting a contact angle close to 90° are generally considered to possess optimal affinity for adsorption at the interface [35]. As shown in Figure 4A, the θ value of MMP alone was 57.66 ± 1.53°. With increasing XG concentration, the θ of the XG-MMP complexes rose progressively, reaching 77.33 ± 1.15° at an XG concentration of 0.4%. These results may indicate that XG enhances the surface hydrophobicity of MMP, improves its affinity towards the oil phase, and thus promotes its adsorption at the oil–water interface.

This observed increase in hydrophobicity can be attributed to two principal factors. First, the binding of XG to hydrophilic groups on the MMP surface reduces their interaction with the aqueous phase, while the hydrophobic hydrocarbon backbone of XG becomes more exposed on the composite surface. Together, these effects elevate the overall macroscopic hydrophobicity of the XG-MMP complexes. Second, the long-chain XG adsorbed on the MMP surface can provide steric hindrance that effectively prevents interparticle aggregation, thereby maintaining dispersion stability even as surface hydrophobicity increases [40]. From a structural perspective, the incorporation of XG likely contributes to the formation of a denser and more continuous network. At the microscopic scale relevant to contact-angle measurement, this structural consolidation can retard the rapid penetration and spreading of water droplets, resulting in the measured increase in contact angle.

The microstructure of the XG-MMP complexes was examined using SEM. As shown in Figure 4A, MMP alone formed large, irregular, and loosely packed aggregates [14]. With increasing XG concentration, the morphology progressively transitioned from particulate aggregates towards a fibrous, network-like architecture. At an XG concentration of 0.2%, a uniform and continuous network was established, exhibiting markedly greater structural density than the control.

To further assess the development of this network in the hydrated state, cryo-SEM was performed on the control and the sample containing 0.4% XG (Figure 4B). Cryo-SEM presented the native distribution of MMP in an aqueous environment, revealing at a 50 µm scale the presence of filamentous spatial connections between MMPs. However, higher-resolution images (20–10 µm) showed that these connections were discontinuous, unevenly distributed, and structurally fragile in the absence of XG. In contrast, the samples with higher XG concentration formed a highly continuous, uniform, and tightly interconnected three-dimensional network. At higher magnification (20–10 µm), XG molecules can be seen filling the pores between MMP skeletons, and columnar connections between network layers are evident, resulting in a denser matrix. Such dense polysaccharide-protein networks have been widely reported in prior studies [41].

Notably, at the molecular scale, protein-polysaccharide networks in aqueous media often exhibit a filamentous appearance [25]. We therefore hypothesise that, in the present system, XG not only adsorbs onto the surface of MMPs but also acts as a “bridge” within the pores of the fibrillar network, cross-linking MMP aggregates into a coherent and stable structure, thereby significantly enhancing the structural integrity and order of the network.

### 3.2. Emulsifying Capacity Analysis

**Figure 5:**
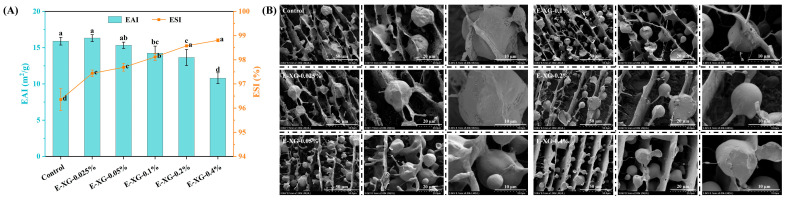
EAI, ESI (**A**), and cryo-SEM images (**B**) of MMP and XG-MMP emulsions. Different lowercase letters (a–d) indicate significant differences between XG concentrations (*p* < 0.05). The error bars represent the standard deviation.

The EAI reflects the ability of proteins or protein-polysaccharide complexes to rapidly adsorb and form an interfacial layer during emulsion formation, while the ESI indicates the resistance of emulsion droplets to aggregation, flocculation, and phase separation [26]. As shown in Figure 5A, both the control and the low-XG-concentration samples exhibited high EAI values. With increasing XG content, the EAI decreased progressively from 16 m^2^·g^−1^ to 11 m^2^·g^−1^ (*p* < 0.05). In contrast, emulsion stability was lowest for the control and increased significantly as the XG concentration rose (*p* < 0.05). This apparent contradiction suggests a shift in the dominant stabilisation mechanism of the system—from rapid interfacial adsorption towards structural reinforcement of the continuous aqueous phase and/or the interfacial film. XG and MMP cooperate to form a highly viscous, three-dimensional network within the aqueous phase, which effectively restricts droplet movement, collision, and coalescence [42].

### 3.3. Emulsion Cryo-SEM Analysis

To further elucidate the mechanism by which MMP and XG-MMP complexes influence emulsion stability, the distribution of the aqueous phase and the oil–water interface in emulsion samples was examined using cryo-SEM. As shown in Figure 5B, in the control emulsion, a fibrous network formed by MMP molecules was clearly visible, exhibiting a curved and porous architecture. At higher magnification, an adherent MMP interfacial layer can be observed on the droplet surface, displaying fine, chain-like entanglements. The aqueous-phase structure of the low-XG sample (0.025%) resembled that of the control. With increasing XG concentration, the overall network in XG-MMP emulsions became progressively denser, and oil droplets adopted an ordered spatial arrangement, being embedded within the network layers formed by XG-MMP composites. Enlarged images reveal more clearly that the thick XG-MMP interfacial layer on the droplet surface has partially detached, likely due to rapid freezing. This observation aligns with the microstructure previously described for the XG-MMP aqueous system, supporting the view that XG molecules infiltrate and fill the native porous structure of MMP. These interfacial layers remain connected to unadsorbed XG-MMP complexes in the aqueous phase, creating a cohesive, continuous network that enhances steric hindrance and electrostatic repulsion between droplets, thereby improving emulsion stability. This mechanism is consistent with the findings of Aljewicz et al. [43], who reported that at high concentrations, xanthan gum intercalates between casein micelles under acidic conditions, filling interstitial spaces and contributing to micellar stabilisation.

### 3.4. Emulsion Particle Size and Zeta-Potential Analysis

**Figure 6:**
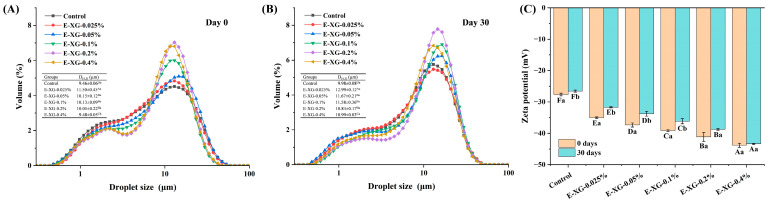
The particle size (at 0 day (**A**) and 30 days (**B**)) and zeta-potential (**C**) for MMP (control) and a series of XG-MMP complexes. The experimental results for D_[4,3]_ are expressed in µm as the mean ± standard deviation. Significant differences among XG concentrations are indicated by distinct uppercase letters (A–F) (*p* < 0.05). Differences in storage time within the same XG concentration are denoted by different lowercase letters (a, b) (*p* < 0.05).

Particle size and zeta-potential are key indicators of emulsion stability. These parameters were measured immediately after preparation (day 0) and after 30 days of storage. As shown in Figure 6A, the initial emulsion without XG (control) displayed a low, broad particle size distribution, reflecting heterogeneous droplet sizes and limited emulsification efficiency. With increasing XG concentration, the breadth of the size distribution did not change markedly, yet a clear bimodal pattern emerged, characterised by a distinct main peak. This suggests that high XG levels do not substantially improve droplet uniformity but instead promote the formation of two distinct droplet populations within the emulsion. In terms of D_[4,3]_ values, the emulsion particle size first increased and then decreased with XG content. The largest D_[4,3]_ value (11.50 µm) occurred at the lowest XG concentration tested (0.025%), after which it gradually declined. Nevertheless, all XG-containing samples exhibited significantly larger droplet sizes than the control (*p* < 0.05).

As shown in Figure 6B, the particle size distribution profiles of all emulsion samples remained highly consistent throughout storage, with no marked shift in shape. Although droplet size increased slightly after 30 d, it still showed a trend of first increasing and then decreasing with the concentration of XG. At the lowest XG concentration (0.025%), the largest value of D_[4,3]_ was recorded, 11.50 µm (*p* < 0.05). The absence of a substantial increase in particle size after prolonged storage suggests that significant coalescence did not occur. This can be explained by the dual stabilising role of XG at higher concentrations, which provides steric hindrance in the aqueous phase while also enhancing electrostatic repulsion between XG-MMP complexes, thereby effectively suppressing droplet collision and aggregation [44]. It is worth noting that even the control sample exhibited no significant growth in particle size after storage. This may imply that the MMP-stabilised oil–water interface remained largely intact over time, or that the self-assembled network formed by MMP in the aqueous phase was sufficient to restrict droplet migration and coalescence in the absence of polysaccharide.

As shown in Figure 6C, in the initial emulsions, the control sample exhibited the lowest absolute zeta-potential, 26.31 mV (*p* < 0.05). With increasing XG concentration, the absolute zeta-potential rose significantly from 35.03 mV to 43.8 mV (*p* < 0.05). After 30 days of storage, the absolute zeta-potential declined in all samples, with the most significant decrease (from 35.03 mV to 31.70 mV) observed for the lowest XG concentration (0.025%) (*p* < 0.05). In contrast, the high-XG (0.4%) emulsion showed no statistically significant reduction (*p* > 0.05). With the exception of the control sample, all XG-containing emulsions maintained an absolute zeta-potential above 30 mV, a value commonly associated with electrostatic stabilisation [45]. In neutral aqueous medium, the anionic character of XG increases the negative charge density at the droplet surface or in the aqueous phase, thereby enhancing electrostatic repulsion between XG-MMP complexes and hindering droplet aggregation [13]. The lower initial zeta-potential of the control sample likely led to its poorer long-term stability. Despite the decrease in zeta-potential after storage, no significant increase in D_[4,3]_ values of the control and low XG emulsions was detected. This suggests that instability in the samples may not follow the classical pathway of extensive flocculation or coalescence, which typically leads to pronounced droplet growth. Instead, weak reversible aggregation or non-coalescent phase separation may be the operative instability mechanisms. Consequently, direct tracking of the emulsion’s macro- and micro-morphology throughout storage is required to clarify this.

### 3.5. Emulsion Appearance and Microstructure Analysis

**Figure 7:**
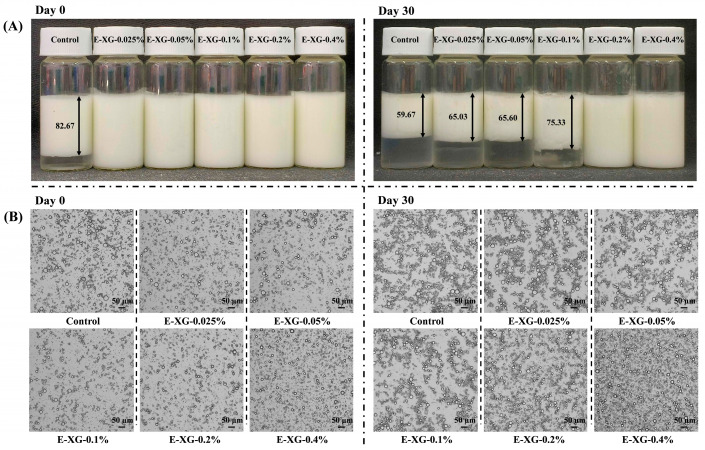
Appearance (**A**) and microscopic images (**B**) for MMP (control) and a series of XG-MMP complexes.

Figure 7A presents the macroscopic appearance of all emulsion samples immediately after preparation (day 0) and following 30 days of storage. Initially, the control sample displayed marked phase separation, reflecting poor stability. After storage, the emulsion-layer height in the control decreased to 59.67% of the initial value, confirming severe destabilisation. Low XG samples also exhibited obvious stratification, with emulsion layer heights of 65.3%, 65.6%, and 75.33% as XG concentration increased. In contrast, emulsions containing 0.2% and 0.4% XG remained visually homogeneous throughout storage, showing no discernible phase separation. This demonstrates that sufficiently high concentrations of XG effectively counteract the inherent stability limitations of MMP-stabilised emulsions [29].

The microstructure of the emulsions was examined by optical microscopy (Figure 7B). Initial droplet sizes were similar across all groups; however, emulsions containing higher XG concentrations displayed a more uniform droplet distribution. After 30 days of storage, distinct aggregates had formed in the control and low-XG samples. These aggregates did not cause widespread droplet coalescence but instead confined and embedded oil droplets within a network of MMP or XG-MMP complexes, leading to localised, discontinuous oil-rich regions. This distinct microstructure is consistent with the particle-size and zeta-potential data presented earlier. It suggests that in emulsion systems with insufficient electrostatic stability, MMPs, whether alone or associated with limited XG, undergo a form of restricted aggregation. This process does not result in direct droplet coalescence but rather entraps oil droplets within a spatially confined aggregate [46]. These aggregates ultimately separate from the continuous phase, causing the observed macroscopic stratification, and may give rise to a local gel-like or weak-gel network microstructure resembling that of an emulsion gel.

### 3.6. Emulsion CLSM Analysis

**Figure 8:**
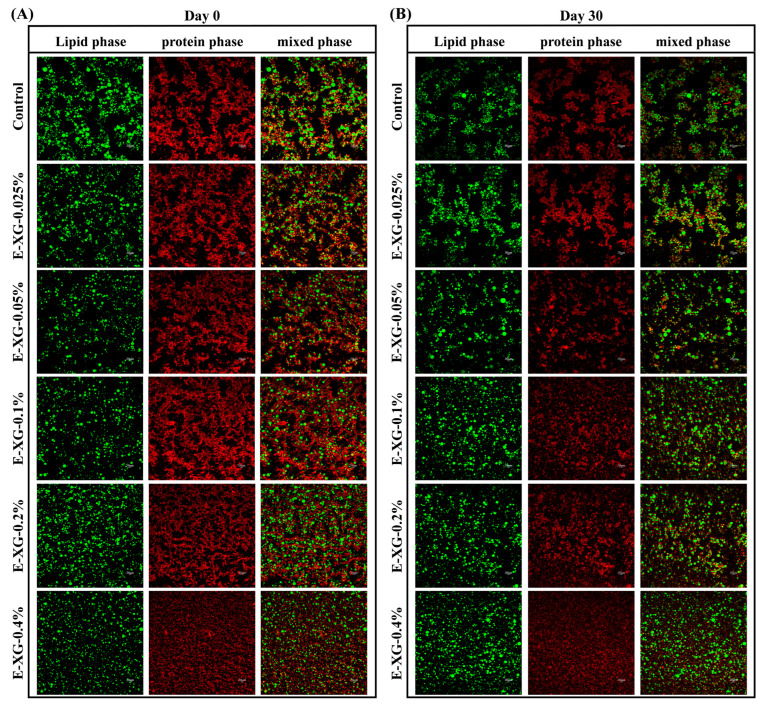
CLSM for MMP (control) and a series of XG-MMP complexes at 0 day (**A**) and 30 days (**B**). The lipid phase is displayed in green and the protein phase is displayed in red.

CLSM further elucidated the evolution of emulsion microstructure with XG concentration. In Figure 8A, lipid droplets are shown in green and MMPs or XG-MMP complexes in red. The droplet size and distribution in the CLSM images are identical to those in the optical microscope images. In the fresh control sample, a continuous red ring surrounding the lipid droplets (red hollow sphere) confirms that MMP particles adsorbed effectively at the oil–water interface, forming a coherent interfacial layer. Beyond this layer, however, abundant free MMP aggregates were present in the aqueous phase. These aggregates are interconnected into extended red domains that encapsulate the interfacial droplets. Viewed through the green channel, lipid droplets were not uniformly dispersed but were confined within discrete regions defined by the MMP network, yielding an overall heterogeneous morphology alternating between lipid-droplet-rich zones and extensive black voids (aqueous, droplet-depleted regions).

Emulsions containing low XG concentrations (<0.2%) displayed a microstructure similar to the control, indicating that at these levels XG did not alter the MMP-dominated architecture in which interfacial adsorption and bulk aggregation coexist. XG molecules likely remained predominantly in the aqueous phase, where they modestly increased viscosity or participated in weak interactions that slightly modified aggregate size or connectivity, without fundamentally shifting the system’s inherent tendency to form localised gel-like networks [29]. These microstructural observations align with the earlier zeta-potential, particle size, and storage-stability data, collectively demonstrating that low XG concentrations could not effectively suppress bulk MMP aggregation and therefore failed to mitigate the local heterogeneity and macroscopic stratification driven by aggregation.

As shown in Figure 8B, after 30 days of storage, the control and low-XG emulsions displayed more extensive aggregates of MMP or XG-MMP complexes, accompanied by a marked expansion of the droplet-depleted aqueous regions. This microstructural evolution corresponds directly to the macroscopic phase separation observed visually. In contrast, emulsions containing 0.2% and 0.4% XG retained a relatively intact morphology, with no evidence of large-scale droplet coalescence or extensive aggregation of XG-MMP complexes.

Notably, although CLSM revealed clear aqueous-phase aggregation, particle-size analysis did not indicate significant overall emulsion aggregation. This discrepancy suggests that the dilution required for particle-size measurement likely dissociated the weakly bound XG-MMP aggregates [47]. The observation further supports the view that these aggregates are stabilised primarily by weak, reversible interactions such as hydrophobic forces and hydrogen bonding. Consequently, CLSM provides a more accurate representation of the true microstructure of the emulsion system under native, undiluted conditions.

### 3.7. Emulsion Rheological Properties Analysis

**Figure 9:**
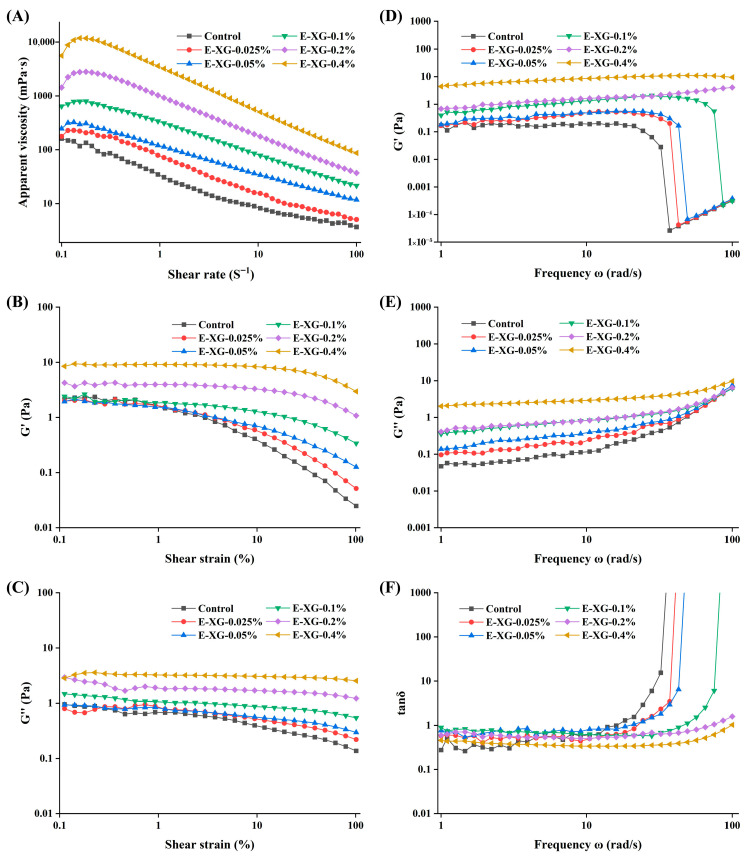
Apparent viscosity (**A**), amplitude scanning (**B**,**C**), and frequency scanning (**D**–**F**) for MMP (control) and a series of XG-MMP complexes.

The steady-shear rheological behaviour of the XG-MMP composite emulsions is presented in Figure 9A. All emulsions exhibited a pronounced decrease in apparent viscosity with increasing shear rate, characteristic of shear-thinning (pseudoplastic) behaviour [48]. As the XG concentration rose, the viscosity increased systematically across the entire shear-rate range. This effect can be ascribed to the nature of XG as a hydrophilic polysaccharide. In aqueous solution, XG chains form a weakened and extended three-dimensional network through hydrogen bonding and chain entanglement [49]. At higher concentrations, intermolecular interactions are reinforced, yielding a denser and more continuous network that enhances the structural integrity of the continuous phase. The network raises internal friction and flow resistance, which is macroscopically observed as an increase in apparent viscosity. Thus, the microstructural modification substantially alters the macroscopic rheological properties and physical stability of the emulsion.

Amplitude sweep tests were performed to determine the linear viscoelastic region (LVR) and to characterise the yielding behaviour of the emulsions. As shown in Figure 9B,C, the amplitude-dependent response clearly illustrates the relationship between the dynamic moduli and applied strain. Within the low strain region (0.1–10%), the storage modulus (G′) consistently exceeded the loss modulus (G″), and both moduli remained essentially constant, confirming that the systems were within the LVR. In this regime, the emulsions behaved as stable, solid-like materials capable of recoverable elastic deformation. As strain increased further, G′ decreased markedly, signalling the gradual breakdown of the microstructure and the transition from linear viscoelastic behaviour to nonlinear flow.

Notably, the critical strain of the emulsions increased substantially with rising XG concentration, demonstrating that higher XG levels enhance the system’s resistance to shear-induced failure. This concentration-dependent strengthening aligns with findings reported by Li et al. [48] for xanthan-gum-stabilised lysozyme-nanoparticle emulsions, confirming that xanthan gum generally improves the mechanical robustness of emulsion systems. This behaviour can be ascribed to the formation of a more resilient and persistent three-dimensional microstructure by XG molecules within the continuous phase. This structured matrix effectively dissipates and resists external shear stresses while restricting droplet motion, thereby enhancing both the macroscopic stability and yield resistance of the emulsion.

The linear viscoelastic response of the emulsions was further characterised through frequency-sweep measurements (Figure 9D,E). The results indicate that both the storage (G′) and loss (G″) moduli increased markedly with rising XG concentration, confirming that XG promotes the development of a more developed microstructure in the continuous phase, thereby enhancing the overall viscoelasticity and structural stability of the system [48]. In the control and low-XG samples (0.025–0.1%), G′ exhibited a pronounced decrease at higher frequencies, suggesting that the limited microstructure formed at these concentrations is more susceptible to breakdown under dynamic shear.

Figure 9F illustrates the frequency dependence of the loss factor (tan δ). In the low-frequency range (1–10 rad·s^−1^), tan δ remained below 1 for all samples, confirming that G′ exceeded G″ and that the systems behaved as predominantly elastic, solid-like materials. As the frequency increased above approximately 37 rad·s^−1^, a marked decrease in G′ was observed for the control and low-XG samples (0.025–0.1%), with G″ surpassing G′. This shift indicates a transition from elastic-dominated to viscous-dominated behaviour. The transition reflects the irreversible breakdown of the microstructure formed by XG and droplets under high-frequency shear, leading to structural collapse and a pronounced change in rheological response. These results demonstrate that emulsions with low XG content possess limited structural integrity under intense dynamic loading [50].

### 3.8. Mechanism Schematic

**Figure 10:**
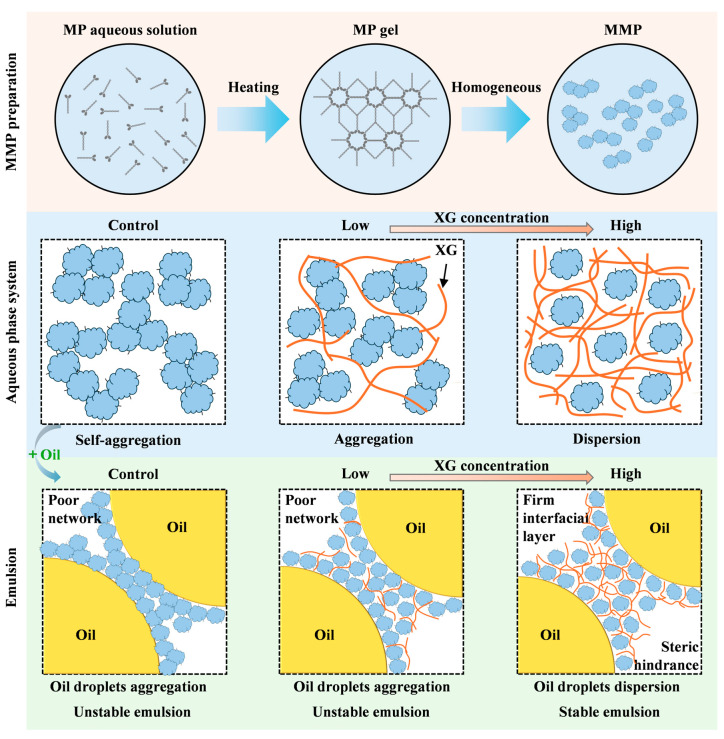
The proposed mechanism diagram for MMP (control) and a series of XG-MMP complexes.

To elucidate the mechanism by which XG stabilises MMP emulsions, Figure 10 proposes a possible underlying mechanism based on the experimental data. MMPs are microgel particles obtained via thermogelation and disruption of MP, retaining the microporous structure of the parent protein. When the XG concentration ranged from 0 to 0.1%, the particle size of the composite system increased and the zeta potential rose slightly, indicating enhanced aggregation of the composite. Although MMP itself possesses surface activity, its hydrophobic regions drive self-aggregation in the aqueous phase; however, low concentrations of XG failed to fully cover these regions and instead promoted the formation of larger aggregates through the extension of molecular chains. The ‘oil-rich droplet zones’ observed microscopically, together with the rheological instability, are direct consequences of these aqueous aggregates displacing the oil droplets. Notably, this aggregation did not disrupt the oil–water interfacial film (CLSM revealed no droplet coalescence); rather, it promoted macroscopic instability via flocculation and gravitational separation, resulting in the formation of a distinct cream layer and a separated aqueous phase.

When the XG concentration reached 0.2%, particle size decreased and the zeta potential increased significantly, indicating that XG had fully coated the MMP surface. The enhanced electrostatic repulsion and steric hindrance effects jointly promoted the separation and uniform dispersion of the composite particles. Furthermore, a uniform and dispersed droplet distribution was observed microscopically, and the rheological results indicated that XG also enhanced the shear resistance of the emulsion. These findings are consistent with those reported by Xie et al. in their study of emulsions stabilised by soy protein isolate-XG complexes: at low XG concentrations, uneven distribution of XG at the oil–water interface may lead to droplet attraction and aggregation, whereas at high XG concentrations, the increased coverage of XG at the interface effectively inhibits droplet aggregation and enhances emulsion stability [51]. Hashemi et al. [52] also explicitly noted that the primary mechanism by which protein-polysaccharide particles stabilise Pickering emulsions involves the irreversible adsorption of particles at the interface, forming a dense interfacial layer, alongside the accumulation of non-adsorbed particles or the formation of a network structure in the continuous phase, as well as depletion stabilisation.

Therefore, based on the above findings, we hypothesise a dual stabilisation mechanism, whereby a fundamental shift in the emulsion stabilisation behaviour occurs once the XG concentration reaches 0.2%: (1) a sufficient quantity of well-dispersed XG-MMP complexes forms a continuous and stable interfacial layer; and (2) the XG-MMP complexes present in the aqueous phase assemble into a gel-like network, effectively confining the interfacially stabilised droplets within their matrix. These two mechanisms act synergistically to suppress droplet migration and gravitational separation, thereby conferring long-term macroscopic stability upon the system.

## 4. Conclusions

This study systematically investigated the influence of XG concentration on the stabilisation mechanism of MMP-based Pickering emulsions. Through a comprehensive analysis of aqueous-phase interactions, oil–water interfacial microstructure, interfacial properties, and rheological behaviour, a critical XG concentration of 0.2% was identified. Below this threshold, the behaviour of the emulsion is governed by aggregation-driven instability; above this threshold, stability is conferred by uniformly dispersed MMP-XG complexes.

In the absence of XG, MMP can effectively adsorb at the interface and prevent droplet aggregation; however, pronounced phase separation still occurs under electrostatic shielding. This observation indicates that when Pickering emulsions are stabilised solely by large, irregular protein microgels, spontaneous hydrophobic aggregation among particles—both in the aqueous phase and at the interface—constitutes the fundamental limitation to long-term stability. XG and MMP primarily interact through electrostatic, hydrogen bonding, and hydrophobic interactions. When the XG concentration is below 0.2%, depletion flocculation of MMP and aggregation of XG-MMP complexes dominate, leading to macroscopic phase separation. Above the critical concentration, enhanced electrostatic repulsion and steric hindrance limit droplet aggregation. Within this concentration range, XG is uniformly dispersed in the continuous phase, thereby spatially restricting the movement of oil droplets. Consequently, droplet migration and aggregation are inhibited, ensuring the long-term stability of the emulsion.

This study demonstrates that by precisely regulating the concentration of structured polysaccharides, the stabilisation mechanism may transition from one relying solely on interfacial adsorption to a dual mode combining interfacial enhancement with aqueous-phase confinement. This finding provides a sound theoretical basis for the application of irregular micron-sized protein particles in practical Pickering emulsion formulations. Nevertheless, this study was conducted under fixed conditions, such as pH and ionic strength, whereas in practical systems, the preparation of Pickering emulsions is often influenced by a variety of environmental factors. Under the interplay of different environmental conditions, the interaction mechanism between MMP and XG may undergo corresponding changes. Therefore, future research should further investigate the regulatory effects of environmental factors, including pH and ionic strength, on the critical transition concentration, with a view to extending the application of this strategy to more complex emulsion systems.

## Data Availability

The original contributions presented in this study are included in the article. Further inquiries can be directed to the corresponding authors.
